# Albumin redox state of maintenance haemodialysis patients is positively altered after treatment

**DOI:** 10.1186/s12882-023-03317-9

**Published:** 2023-09-18

**Authors:** Kristina Boss, Margret Paar, Katja Waterstradt, Kerstin Schnurr, Philipp Ickerott, Ulrike Wieneke, Ralf Spitthöver, Karl Oettl, Andreas Kribben

**Affiliations:** 1 Department of Nephrology, University Hospital Essen, University Duisburg-Essen, 45147 Essen, Germany; 2 Division of Medicinal Chemistry, Otto Loewi Research Center, Medical University of Graz, Graz, Austria; 3 MedInnovation GmbH, Berlin, Germany; 4 Dialyse- und Lipid-Zentrum Nordrhein, Essen, Germany; 5 Gemeinschaftspraxis für Nieren- und Hochdruckkrankheiten Essen-Steele, Essen, Germany

**Keywords:** Albumin redox state, Haemodialysis, Human non-mercaptalbumin, High flux

## Abstract

**Background and Aim:**

Maintenance haemodialysis patients have increased morbidity and mortality which is mainly driven by an elevated inflammation level due to the uraemic milieu. A major part of this increased inflammation level is the degree of oxidative stress which can be assessed by albumin redox state (ARS). Aim of this study was to evaluate how the ARS is affected by a haemodialysis treatment and different dialyzer properties.

**Methods:**

ARS was determined before and after haemodialysis treatment by fractionating it into reduced human mercaptalbumin (HMA), reversibly oxidized human non-mercaptalbumin 1 (HNA-1), and irreversibly oxidized human non-mercaptalbumin 2 (HNA-2) by high-performance liquid chromatography. In healthy individuals, albumin circulates in the following proportions: HMA 70–80%, HNA-1 20–30% and HNA-2 2–5%. High flux (FX 100 [Fresenius Medical Care], BG 1.8 [Toray], Xevonta Hi 18 [B. Braun], CTA-2000 [Kawasumi]) and low flux FX10 [Fresenius Medical Care] dialyzers were used.

**Results:**

58 patients (59% male, median age 68 years, median time on haemodialysis 32 month) were included in the study. Before haemodialysis treatment, HMA (median 55.9%, IQR 50.1–61.2%) was substantially lower than in healthy individuals. Accordingly, oxidized albumin fractions were above the level of healthy individuals (median HNA-1 38.5%, IQR 33.3–43.2%; median HNA-2 5.8%, IQR 5.1–6.7%). Before haemodialysis treatment HMA was significantly higher in patients usually treated with high flux membranes (p < 0.01). After haemodialysis treatment there was a significant increase of HMA and a decrease of HNA-1 and HNA-2 (p < 0.01). These effects were more pronounced in patients treated with high flux dialyzers (p < 0.01). There were no differences of ARS alteration with regard to the dialyzer´s sterilization mode or the presence of diabetes.

**Conclusion:**

The study confirms that the ARS is positively altered by haemodialysis and shows for the first time that this effect depends on dialyzer properties.

**Supplementary Information:**

The online version contains supplementary material available at 10.1186/s12882-023-03317-9.

## Introduction

Kidney diseases have a major effect on global health, as more than 850 million of patients worldwide have kidney diseases, which are a direct cause of global morbidity and mortality [1]. In patients with end-stage kidney disease (ESKD), haemodialysis is the predominant form of kidney replacement therapy, which is carried out in about 2.8 million patients worldwide [2]. Haemodialysis patients have an all-cause annual mortality of 16% [3, 4]. This is mainly driven by an elevated inflammation level in these patients due to the uraemic milieu, which consecutive leads to increased cardiovascular morbidity [5]. A major part of this increased inflammation level is the degree of oxidative stress. Oxidative stress is defined as disturbances in the pro-/antioxidant balance, which cause several complications in ESKD patients, e.g., hypertension, atherosclerosis, and anaemia via high amounts of reactive oxygen (ROS) and nitrogen (RNS) species [6].

An endogenous mechanism to counter oxidative stress is the use of albumin as a scavenger for ROS and similar molecules. Albumin contains the largest source of extracellular thiols, since it has a reduced cysteine residue [7]. This characteristic makes it a suitable target structure for assessing the degree of oxidative stress by evaluation of albumin redox state (ARS), which can be defined in different terms. Here we understand it as the redox state based on cysteine-34 (Cys-34) which may occur in three different variants: (i) in a fully reduced form as thiol as human mercaptalbumin (HMA), (ii) in a mild reversibly oxidized form as a disulfide mainly with another cysteine as human nonmercaptalbumin-1 (HNA-1) and (iii) irreversibly oxidized to the sulfinic or sulfonic acid form, human nonmercaptalbumin-2 (HNA-2). The degree of albumin oxidation in general and the proportion of irreversibly oxidized albumin (HNA-2) in particular, can be an indicator of the oxidative stress level of the patient. Also, it may have impact on the functional properties of the albumin molecule [8, 9]. In haemodialysis patients, the level of oxidized albumin fractions is a direct positive predictor of mortality [10].

Since the 1980s it is known, that haemodialysis alters albumin redox state [11]. Several studies evaluated a variety of substances and techniques for using albumin redox potential to reduce oxidative stress [12–14]. In these studies, the effect of haemodialysis on the level of oxidative stress was investigated in small cohorts in which a mix of different dialyzers was used. Time point of blood sample drawing as well as redox markers investigated also varied. Thus, it is still unclear to what extent the ARS can be influenced by specific selection of the dialyzer.

The aim of this study was to investigate how ARS is affected by a haemodialysis treatment in general and by different dialyzer properties, namely dialyzer material, sterilization mode of the dialyzer, and ultrafiltration coefficient in particular.

## Methods

### Study design and patient enrolment

The study was conducted in the Department of Nephrology of the University Hospital Essen and in two cooperating outpatient dialysis centers in Essen, Germany in September 2022. Inclusion criteria were age ≥ 18 years, ability to provide informed consent, and maintenance haemodialysis for at least 6 months before inclusion in the study. Exclusion criteria were age < 18 years and/or no declaration of consent. All patients enrolled in the study underwent baseline laboratory studies. Albumin quantity and albumin fractions were measured before and after haemodialysis treatment. Blood samples were collected from the arterial side of the arteriovenous fistula immediately before and after the haemodialysis.

### Haemodialysis treatment properties

Haemodialysis treatment was carried out according to Dialysis Standard of the German Society of Nephrology [15]. Each patient received 4.0 h haemodialysis 3x/week with the same treatment mode, dialyzer, and anticoagulation for at least 6 months before inclusion in the study. All patients had no dialysis treatment in the two days prior to blood collection. High flux (FX 100 Fresenius Medical Care, Bad Homburg v.d.H, Germany; BG 1.8 Toray Medical, Tokia, Japan; Xevonta Hi 18 B. Braun, Melsungen, Germany; CTA-2000 Kawasumi, Japan) and low flux (FX10 Fresenius Medical Care, Bad Homburg v.d.H, Germany) dialyzers were used. High flux dialyzers were used for haemodialysis treatment only. Blood flow was set to more than or equal to 200 mL/min; dialysate flow was set to more than or equal to 500 mL/min. Blood and dialysate flow were comparable between centers and apply for all treatments.

### Baseline laboratory measurements

To exclude relevant confounding conditions, baseline laboratory measurements were performed. Variables associated with systemic infection, white blood cell count (WBC), high sensitivity C-reactive protein (hsCRP) and procalcitonin (PCT), were not elevated compared to the normal range of the parameter for healthy subjects (Supplement Table 1). Baseline laboratory measurements were performed by Central Laboratory of University Hospital Essen, Essen, Germany. Albumin quantity was determined using Albumin Bromocresol Green (BCG) Assay Kit (Abcam) by UV-Vis spectrometry. To avoid systematic overestimation of albumin quantity after haemodialysis treatment, the albumin concentration was corrected for ultrafiltration-induced haemoconcentration according to the formula by Schneditz et al. [16].

### High-performance liquid chromatography

The redox state of plasma albumin was determined by fractionating it into human mercaptalbumin (HMA), human nonmercaptalbumin-1 (HNA-1), and human non-mercaptalbumin-2 (HNA-2) with high-performance liquid chromatography (HPLC), as described by Oettl et al. [8]. In healthy humans, albumin predominantly (70–80%) circulates in a reduced state (HMA), with a free thiol group in the Cys-34 residue acting as a free radical scavenger for reactive oxygen and nitrogen species. Also, lower amounts of HNA-1 (20–30%), the reversibly oxidized state of albumin, and HNA-2, the irreversibly oxidized state, circulate in the blood (2–5%) [8]. Interpretations of the results of this study refers to these albumin redox state proportions. No measurements of the proportion of albumin redox fractions in healthy subjects were carried out in the framework of this study.

### Statistical analysis

All statistical analyses and graphical evaluations were performed with GraphPad Prism software (version 9.4; San Diego, CA, USA) and SAS (version 9.4; Cary, NC, USA). The Kolmogorov-Smirnov test was used for normality testing. Paired or unpaired two-tailed Students t-tests were used for comparison of means. Statistical significance was set at the level of *P* < 0.05. The Welch´s extension to the t-test was used to address unequal variances and/or unequal sample sizes that occurred in some comparisons.

### Ethics approval

The study was performed in accordance with the Declaration of Helsinki and the International Conference on Harmonization Good Clinical Practice guidelines. The study was approved by the local ethics committee of the University of Duisburg-Essen (22-10806-BO).

## Results

### Patient and treatment characteristics

A total of 58 patients participated in this study. 34 of the 58 patients (59%) were men. The median age was 68 years (range 27–89 years). The most common renal diseases in the study population were hypertension (38 of 58 patients, 66%) and diabetes mellitus (18 of 58 patients, 31%). Almost all patients had an arteriovenous fistula as dialysis access (53 of 58 patients, 91%). The median time on haemodialysis was 32 months (IQR 17–47 months). 57 patients received heparin; 1 patient received argatroban based anticoagulation. Detailed patient and dialyzer characteristics are presented in Tables 1 and 2.

**Table 1:** Patient characteristics

Characteristic	All n = 58	Polysulfone low flux n = 32	Polysulfone high flux n = 16
Demographic data			
Age [years], median (range)	68 (27–89)	70 (37–84)	64 (27–89)
Men, n [%]	34 (59)	18 (56)	11 (69)
Women, n [%]	24 (41)	14 (44)	5 (31)
Weight before treatment [kg], median (range)	83 (58–145)	84 (59–145)	79 (58–140)
BUN before treatment [mg/dl], median (IQR)	60.8 (53.4–68.7)	63.7 (52.5–69.4)	58.8 (52.3–65.6)
Ultrafiltration [L], median (range)	2.4 (+ 1.1 - -4.5)	2.3 (+ 1.1- -4.3)	1.8 (0.8–3.3)
Renal disease, n [%]			
Nephrosclerosis due to hypertension	38 (66)	26 (81)	7 (44)
Diabetic nephropathy	18 (31)	14 (44)	3 (19)
Autoimmune diseases (IgA nephropathy, SLE, AAV)	15 (26)	11 (34)	1 (6)
ADPKD	3 (5)	0	1 (6)
Unknown	4 (7)	0	4 (25)
Other	16 (27)	7 (22)	3 (19)

Abbreviation: AAV Anti-neutrophil cytoplasmic antibody (ANCA)-associated vasculitis, ADPKD Autosomal dominant polycystic kidney disease, SLE Systemic lupus erythematosus. BUN blood urea nitrogen, reference range 6-19.8 mg/dL. IQR interquartile range. The total number of renal diseases exceed 100% since several patients suffered from more than one renal disease. The patient characteristics are not equally distributed between the groups

**Table 2:** Dialyzer characteristics

Dialyzer	n	Material	Sterilization Mode	Ultrafiltration coefficient	PVP
FX 10	32	Polysulfone	Inline steam	Low flux	+
FX 100	6	Polysulfone	Inline steam	High flux	+
Xevonta 18 Hi	10	Polysulfone	Gamma irradiation	High flux	+
BG 1.8	6	PMMA	Gamma irradiation	High flux	non
CTA 2000	4	CTA	Gamma irradiation	High flux	non

Abbreviation: PMMA polymethylmethacrylate, CTA cellulose triacetate, PVP Polyvinylpyrrolidone

### Albumin quantity

**Fig. 1:**
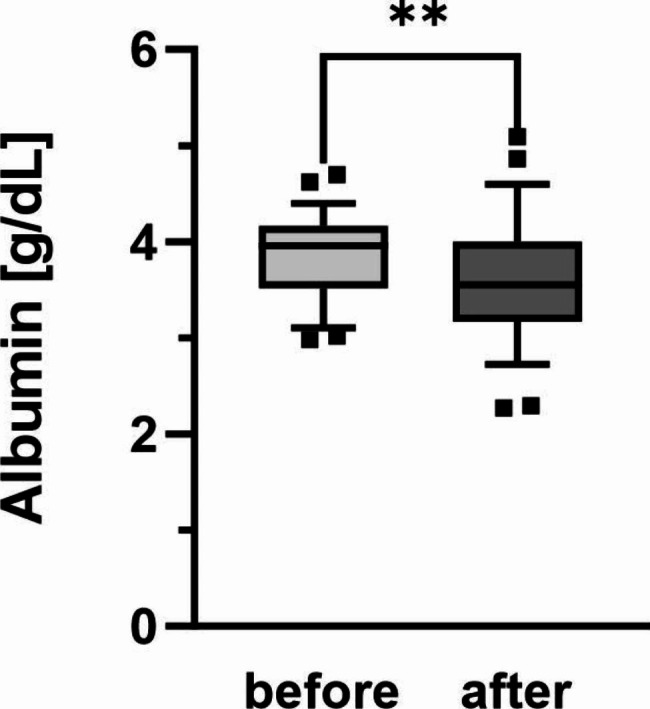
Albumin quantity before and after haemodialysis treatment. Boxes show serum albumin content of n = 58 patients. Central lines denote median values, and upper and lower borders represent 25th and 75th percentiles. The whiskers represent 5–95 percentile. Highest and lowest values are marked as dots. Asterisks mark differences between both groups at a significance level of p < 0.01 with paired Student’s t-test

Albumin quantity was determined before and after haemodialysis treatment. The median albumin quantity before haemodialysis treatment was 3.9 g/dL (IQR 3.5–4.2 g/dL). After haemodialysis treatment, the median albumin quantity decreased to 3.5 g/dL (IQR 3.2-4.0 g/dL; p = 0.0012, Fig. 1). Before haemodialysis treatment, six of 58 patients showed hypoalbuminemia. In two of them, albumin quantity was within the normal range after haemodialysis treatment. 17 patients showed new hypoalbuminemia after the haemodialysis treatment, of which 12 patients were treated with a high flux dialyzer. The median albumin quantity of these 17 patients after haemodialysis treatment was 3.06 g/dL (IQR 2.9–3.3 g/dL). The median albumin loss after haemodialysis treatment in these patients was 0.7 g/dL (IQR 0.5–1.2 g/dL).

### Albumin redox state – total cohort

**Fig. 2:**
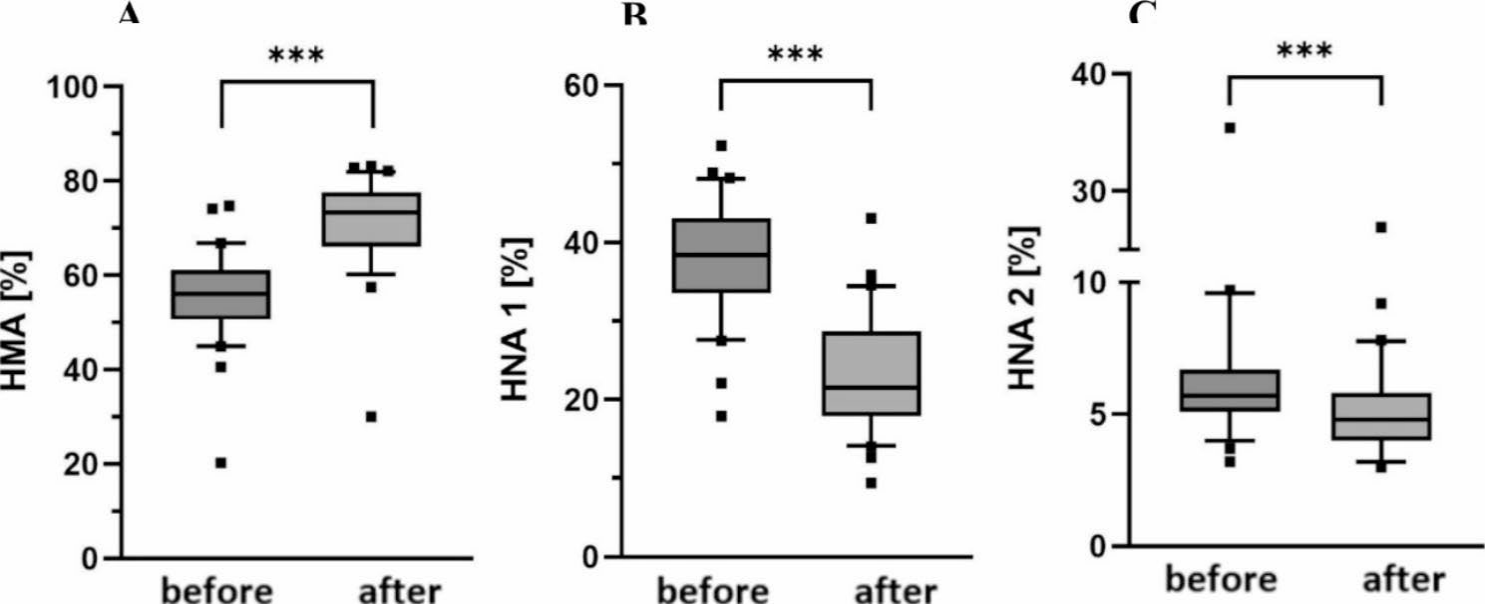
Albumin redox state before and after haemodialysis treatment. Boxes show **(A)** human mercaptalbumin fraction (HMA), **(B)** human nonmercaptalbumin-1 (HNA-1) and **(C)** human nonmercaptalbumin-2 (HNA-2). Data include n = 58 patients. Central lines denote median values, and upper and lower borders represent 25th and 75th percentiles. The whiskers represent 5–95 percentile. Highest and lowest values are marked as dots. Asterisks mark differences between both groups at a significance level of p < 0.001 with paired Student’s t-test

Albumin redox state was determined before and after haemodialysis treatment. In the total cohort, the proportion of reduced HMA was with median 56.0% substantially lower than in healthy individuals. Accordingly, the proportions of reversibly oxidized HNA-1 and irreversibly oxidized HNA-2 were higher than in healthy individuals with 38.5% and 5.8%, respectively [8]. After haemodialysis treatment, albumin redox state was significantly shifted to the more reduced state concerning all albumin fractions (p < 0.01, Fig. 2; Table 3). The latter were subsequently in the median range of healthy individuals. Nevertheless, in 25 patients (43%) the proportion auf HMA was still below 70% and in 26 patients (45%) the proportion of HNA-2 was still > 5% after treatment. Albumin redox proportions did not differ between men and women or in terms of age. There were 18 patients with diabetes in this study, 15 of them treated with a FX10 dialyzer. We compared ARS proportions before and after haemodialysis treatment to the 18 patients also treated with a FX10 dialyzer but not suffering from diabetes. There were no significant differences between these two subgroups (Supplement Fig. 1).

**Table 3:** Albumin fractions before and after haemodialysis treatment

	HMA					HNA-1				HNA-2				
All	n = 58					n = 58				n = 58				
Before Treatment*	56.0 50.1–61.2					38.5 33.3–43.2				5.8 5.1–6.7				
After Treatment*	71.6 65.4–76.8					23.2 19.1–28.9				4.8 4.2–5.8				
Dialyzer Material	Polysulfone n = 48		PMMA n = 6		CTA n = 4	Polysulfone n = 48	PMMA n = 6		CTA n = 4	Polysulfone n = 48	PMMA n = 6			CTA n = 4
Before treatment	55.4 50.0-61.1	59.3 55.4–62.6			57.2 49.5–65.0	38.6 33.1–43.6	35.9 33.3–39.3		37.7 29.7–44.9	6.0 5.3–6.9	4.9 4.1–6.2			5.4 5.0-5.7
After treatment	70.4 64.8–76.1	76.2 72.1–76.9			79.1 70.2–80.8	25.1 19.8–29.9	19.8 19.0-22.9		16.1 14.9–25.9	4.9 4.3–5.8	4.6 3.6–5.2			4.5 3.8-5.0
Sterilization method	Gamma n = 20			Inline steam n = 38		Gamma n = 20		Inline steam n = 38		Gamma n = 20			Inline steam n = 38	
Before treatment	59.4 56.2–64.6			53.2 49.0-58.6		36.0 30.7–38.9		40.2 34.9–44.1		5.6 4.6–5.9		6.2 5.5–7.3		
After treatment	76.7 73.2–80.3			68.4 62.4–74.3		19.5 15.4–21.0		26.4 20.6–31.2		4.4 3.6–4.8		5.4 4.5–5.9		
Ultrafiltration coefficient	High Flux n = 26			Low Flux n = 32		High Flux n = 26		Low Flux n = 32		High Flux n = 26		Low Flux n = 32		
Before Treatment**	59.3 56.3–63.3			51.9 48.9–57.2		35.4 30.9–38.2		41.1 36.8–44.3		5.5 4.5-6.0		6.3 5.6–7.3		
After Treatment**	76.7 73.6–80.4			67.3 61.7–72.6		19.5 15.4–21.4		27.5 22.2–31.7		4.4 3.7–4.9		5.5 4.6-6.0		

Table shows albumin fractions according to redox state (%), median and interquartile range (IQR). Abbreviations: HMA human mercaptalbumin, HNA-1 human non-mercaptalbumin 1, HNA-2 human non-mercaptalbumin 2, PMMA polymethylmethacrylate, CTA cellulose triacetate

* Fractions of HMA, HNA-1 and HNA-2 significantly altered after treatment; p < 0.001

** Fractions of HMA, HNA-1 and HNA-2 significantly differ between high/low flux dialyzers; p < 0.001

### Albumin redox state – impact of dialyzer materials

There were dialyzers out of three different materials used in the study. The majority were polysulfone dialyzers (83%), but some patients were also treated with PMMA dialyzers (10%) and CTA dialyzers (7%). There was a trend for lower proportions of oxidized albumin forms in patients treated with PMMA or CTA dialyzers, but the meaning of this trend is limited by the small number of patients in these groups.

### Albumin redox state – impact of sterilization mode

**Fig. 3:**
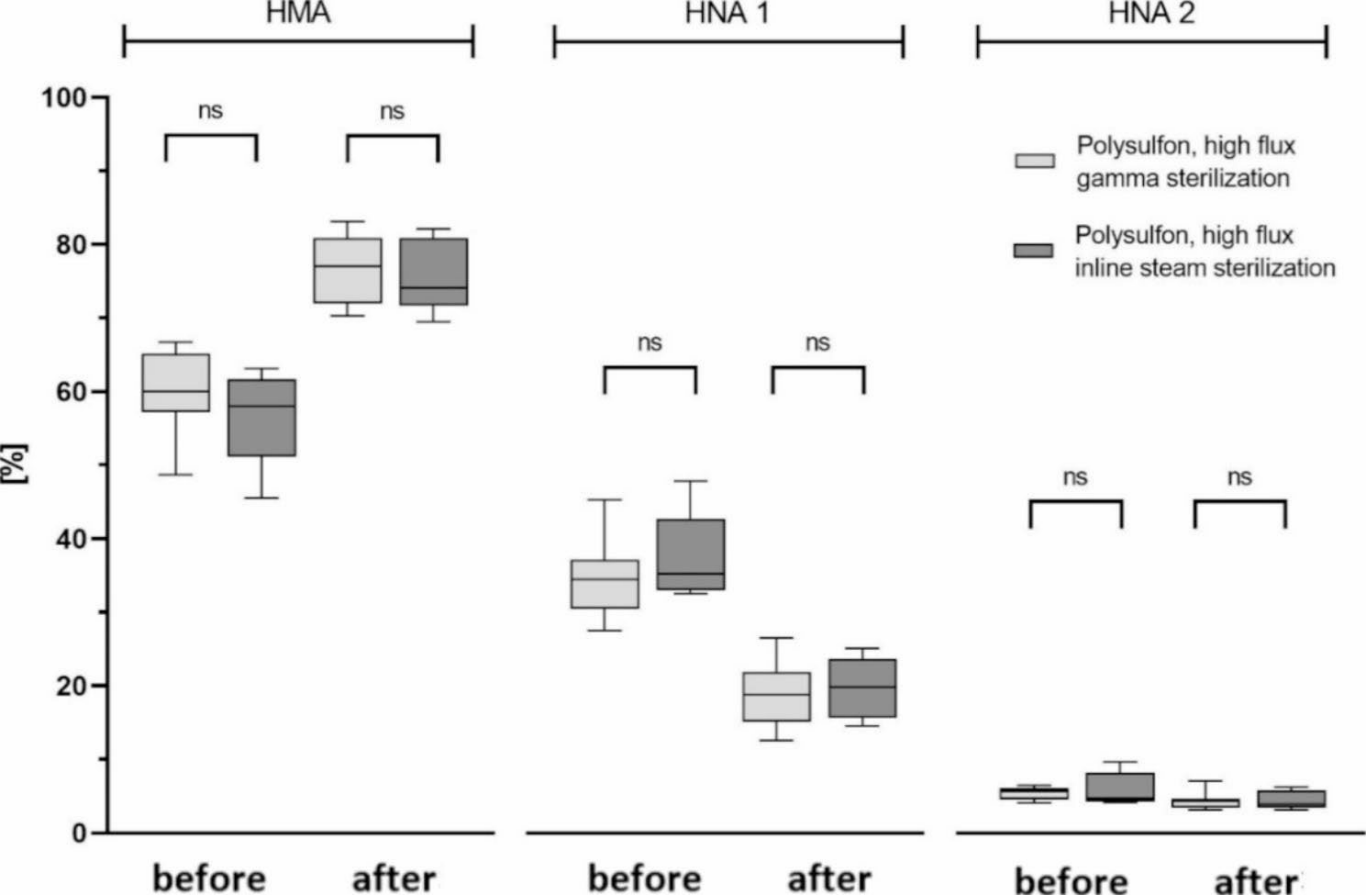
Albumin redox state in relation to dialyzer´s sterilization mode. Boxes show human mercaptalbumin fraction (HMA), human nonmercaptalbumin-1 (HNA-1) and human nonmercaptalbumin-2 (HNA-2) before and after haemodialysis patients. Data include n = 15 patients (gamma sterilization subgroup n = 10, inline steam sterilization subgroup n = 5). Central lines denote median values, and upper and lower borders represent 25th and 75th percentiles. The whiskers represent 5–95 percentile. Highest and lowest values are marked as dots

Different sterilization procedures were applied to the dialyzers used in this study: 38 out of 58 (66%) of the dialyzers have been sterilized by inline steam technology, the other 20 dialyzers (34%) used have been sterilized by gamma irradiation. When comparing polysulfone high flux dialyzers sterilized with gamma irradiation to those sterilized with inline steam technology, there were no significant differences on albumin redox state proportions before and after the haemodialysis treatment (Fig. 3).

### Albumin redox state – impact of ultrafiltration coefficient

**Fig. 4:**
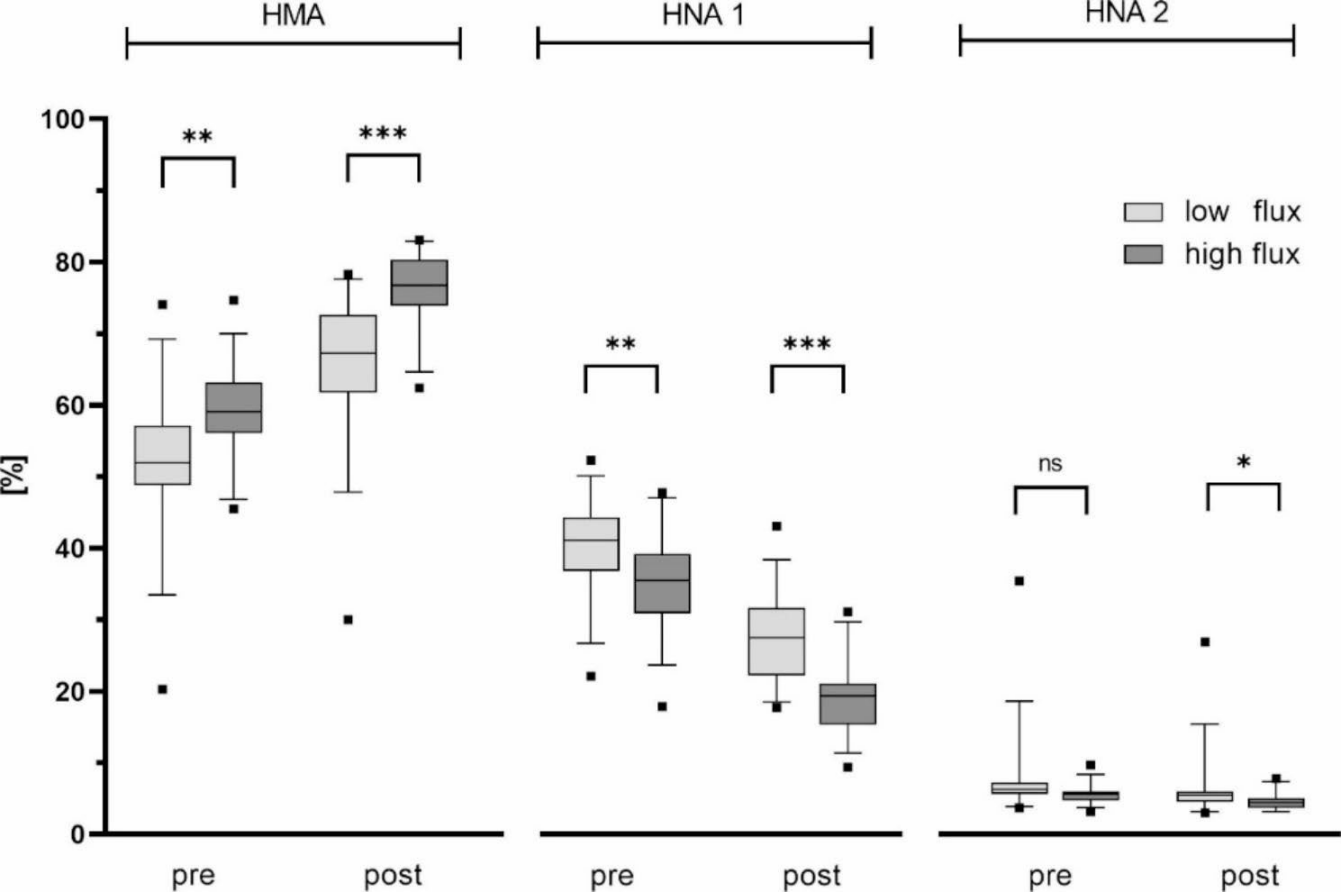
Albumin redox state in relation to dialyzer’s ultrafiltration coefficient. Boxes show human mercaptalbumin fraction (HMA), human nonmercaptalbumin-1 (HNA-1) and human nonmercaptalbumin-2 (HNA-2) before and after haemodialysis patients. Data include n = 58 patients (low flux group n = 32, high flux group n = 26). Central lines denote median values, and upper and lower borders represent 25th and 75th percentiles. The whiskers represent 5–95 percentile. Highest and lowest values are marked as dots. Asterisks mark differences between both groups at a significance level of p < 0.001 with unpaired Student’s t-test

**Fig. 5:**
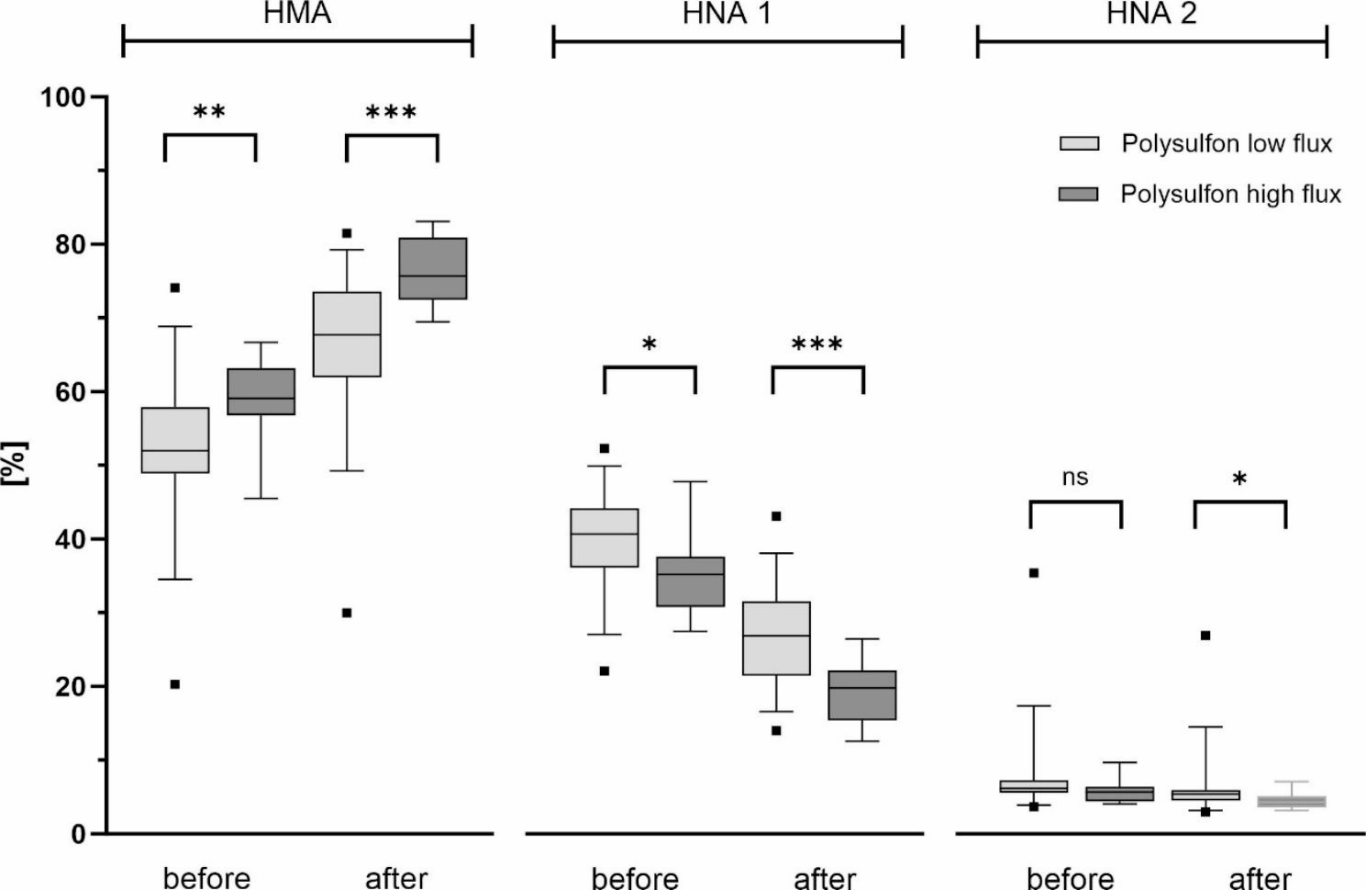
Albumin redox state in relation to dialyzer’s ultrafiltration coefficient – polysulfone subgroup. Boxes show human mercaptalbumin fraction HMA, human nonmercaptalbumin-1 HNA-1 and human nonmercaptalbumin-2 HNA-2 before and after haemodialysis patients. Data include n = 48 patients (Polysulfone low flux group n = 32, Polysulfone high flux group n = 16). Central lines denote median values, and upper and lower borders represent 25th and 75th percentiles. The whiskers represent 5–95 percentile. Highest and lowest values are marked as dots. Asterisks mark differences between both groups at a significance level of p < 0.05 with Welch´s test

Nearly one half of the patients was treated with a high flux dialyzer, a low flux dialyzer, respectively. Patients treated with a low flux dialyzer showed significantly lower proportions of HMA and higher proportions of HNA-1 before haemodialysis treatment (p < 0.01). After haemodialysis treatment, the proportion of oxidized albumin fractions in patients, in which a high flux dialyzer was used, was significantly lower than in patients, in which a low flux dialyzer was used (p < 0.01, p < 0.05 respectively, Fig. 4). Further, all patients with a HMA proportion still < 70% after haemodialysis treatment have been treated with a low flux dialyzer. These effects were also observed when comparing ARS between high and low flux dialyzers all made of polysulfone (p < 0.05, p < 0.01, Fig. 5).

## Discussion

### Key findings

This study evaluated the effect of a haemodialysis treatment in general and of different dialyzer properties in particular on albumin redox state in terms of HMA, HNA-1 and HNA-2 fractions in maintenance haemodialysis patients. Before haemodialysis treatment, HMA fraction was substantially lower than in healthy individuals, oxidized albumin fractions were above the level of healthy individuals [8]. After haemodialysis treatment there was a significant increase of HMA, a decrease of HNA-1 and HNA-2 respectively. These effects were significantly more pronounced in patients treated with high flux dialyzers. There was a tendency for lower fractions of oxidized albumin after haemodialysis treatment with a PMMA or CTA dialyzer compared to polysulfone dialyzers. There were no differences of albumin redox state alteration with regard to the dialyzer´s sterilization mode or the presence of diabetes.

### Comparison with previous studies and future prospects

We observed alterations of albumin quantity after haemodialysis treatment in both directions. Notably, 17 patients showed new hypoalbuminemia after the haemodialysis treatment; most of them were treated with a high flux dialyzer. Extent and impact of albumin loss in the context of haemodialysis is controversially discussed in the literature [17, 18]. Albumin leakage across high flux membranes is known to be a little bit higher than across low flux membranes. The amount of albumin loss we detected in this study is within the range reported in the literature [17]. Whether this is problematic or beneficial for the patients is not completely clear. We are not aware of any study concerning the quality of the albumin lost during treatment.

Several studies evaluated the interaction between haemodialysis treatment and the level of oxidative stress. Soejima et al. pointed out that a haemodialysis treatment increases the patient’s redox buffer by conversion of HNA-1/HMA [19]. In our study we could confirm this effect. After haemodialysis treatment, there was a significant increase of HMA and a decrease of HNA-1, respectively. Soejima et al. concluded, that persistent hypoalbuminemia worsens serum-scavenging activity in maintenance haemodialysis patients, and contributes to the development of cardiovascular and atherosclerotic complications [19]. This relationship was further investigated by Terawaki et al. who showed that the odds for cardiovascular events and cardiovascular death in a 2-year follow up is higher for patients with low HMA before and after haemodialysis treatment than for patients with albumin fractions similar to healthy individuals [20].

To our knowledge, this is the first study that could show that the high interindividual differences of albumin fractions between haemodialysis patients are at least partly due to the dialyzer type used. Patients treated with a low flux dialyzer had significantly lower amounts of HMA before treatment and showed a lower HMA/HNA-1 conversion after haemodialysis treatment than patients treated with a high flux dialyzer. Several techniques and treatment modifications to alter albumin redox state have been discussed in the literature in the last decades [12–14]. More recently, Shigematsu at al. showed in a cross over design that predilution on-line hemodiafiltration improved albumin redox state in maintenance haemodialysis patients compared to using a super high flux dialyzer [21]. It is not yet clear whether the influence on the albumin redox state is brought about by the ultrafiltration coefficient on the one hand and the dialysis mode (haemodialysis vs. haemodiafiltration) on the other hand. In our study, we observed a clear advantage of high flux dialyzers with regard to the proportion of oxidized albumin. This could also result in a consecutive survival advantage for these patients, as Abe et al. demonstrated in their current cohort study [22]. This is in line with the results of a randomized controlled trial by Locatelli et al. [23]. A significant survival benefit was observed among patients with serum albumin ≤ 4 g/dL treated with a high flux membrane. Also, the HEMO study showed positive long-term effects of high flux dialyzer [24].

Further, in our study we found no significant difference of the albumin redox state due to the dialyzer sterilization mode or the presence of diabetes. Dialyzer sterilization mode and material are two interconnected aspects in terms of biocompatibility of dialyzers. Here, polysulfone-based and other dialyzers were evaluated. There was a tendency for lower HNA-1/HNA-2 fractions after haemodialysis treatment with a PMMA or CTA dialyzer compared to polysulfone dialyzers. These results are limited by the low number of patients in these subgroups, but in line with the findings of a larger prospective observational trial by Donati et al. [25]. Further, the PMMA and CTA based dialyzers do not contain PVP. The content and elution of PVP to the patient´s blood, which has been discussed as a possible cause for adverse reactions rarely occurring with synthetic membranes, differ between commercially available dialyzers and were found to be linked to the membrane material and sterilization method [26]. Therefore, the effect of the membrane material itself and a possible impact of hydrophilic modifications on alteration of albumin redox state have to be investigated in further studies. Our study is the first one that showed that certain dialyzer properties can reduce the level of oxidized albumin fractions and thereby possibly could contribute to lowering mortality in maintenance haemodialysis patients.

In conclusion, prospective cross over trials with different dialysis treatment modes and different dialyzer types like the one planned by Donati et al. are needed to further evaluate to what extend dialyzer properties can reduce the level of oxidative stress [27].

### Strengths and Limitations

Our study has several strengths and limitations. To our knowledge, this is the first study to evaluate the effect of different dialyzer properties on albumin redox state in maintenance haemodialysis patients. For this, two different redox-markers, a reversible and an irreversible oxidation, of the same molecule have been investigated. The results are limited by the monocentric design, which is only partly compensated by participating of two different outpatient dialysis centers of the same area. Another limitation is the small number of patients in some subgroups. Further, the number of PMMA and CTA dialyzers used in the study is too small to make more concrete statements about the effect of the dialyzer material on ARS. Due to the study design, some uncertainty remains as to whether the use of different dialyzer per se would lead to the ARS differences observed in this study. Therefore, the results of this study need to be validated by an intervention study, e.g., with a cross-over design.

### Conclusion

The study confirms that the ARS is positively altered by haemodialysis and shows for the first time that this effect depends on dialyzer properties.

### Electronic supplementary material

Below is the link to the electronic supplementary material.

Supplementary Material 1

## Data Availability

The data underlying this article will be shared on reasonable request to the corresponding author.

## Abbreviations

AAV: Anti-neutrophil cytoplasmic antibody (ANCA)-associated vasculitis
ADPKD: Autosomal dominant polycystic kidney disease
ALT: Alanine aminotransferase
AST: Aspartate aminotransferase
CTA: Cellulose triacetate
BUN: Blood urea nitrogen
ESKD: End-stage kidney disease
HMA: Human mercaptalbumin
HNA-1: Human nonmercaptalbumin-1
HNA-2: Human nonmercaptalbumin-2
HPLC: High-performance liquid chromatography
hsCRP: High sensitivity C-reactive protein
LDH: Lactate dehydrogenase
LMWH: Low molecular weight heparin
PCT: Procalcitonin
PMMA: Polymethylmethacrylate
RNS: Reactive nitrogen species
ROS: Reactive oxygen species
SLE: Systemic lupus erythematosus
WBC: White blood cell count
