# Supplementary material for: Albumin redox state of maintenance haemodialysis patients is positively altered after treatment
Source: BMC Nephrol. 2023 Sep 18;24:273. doi: 10.1186/s12882-023-03317-9 (PMC10506191; doi:10.1186/s12882-023-03317-9)
Supplement: Supplementary file 1 — Supplementary Material 1 [file 12882_2023_3317_MOESM1_ESM.pdf]

Supplement

Supplement Table 1: Baseline laboratory measurements

Baseline laboratory measurement	n = 58
**WBC [× 10^9/l]**<br>(normal, 3.6-9.2)	6.9<br>(5.8-8.0)
**Haemoglobin [g/dl]**<br>(normal, 13.7-17.7)	12.0<br>(10.9-12.7)
**Haematocrit [l/l]**<br>(normal, 0.4-0.5)	0.37<br>(0.35-0.38)
**Platelets [× 10^9/l]**<br>(normal, 140-320)	179<br>(143-230)
**Sodium [mmol/l]**<br>(normal 136-145)	139<br>(137-141)
**Potassium [mmol/l]**<br>(normal 3,5-5,1)	5.1<br>(4.6-5.9)
**Calcium [mmol/l]**<br>(normal 2,08-2,65)	2.2<br>(2.1-2.3)
**Ionic Calcium [mmol/l]**<br>(normal 1,15-1,29)	1.04<br>(0.97-1.13)
**Magnesium [mmol/l]**<br>(normal **0,66-1,07**)	0.9<br>(0.8-0.9)
**Phophate [mg/dl]**<br>(normal 2,7-4,5)	5.3<br>(4.3-6.1)
**Serum creatinine [mg/dl]**<br>(normal 0,51-0,95)	7.7<br>(6.5-10.5)
**Uric acid [mg/dl]**<br>(normal 2,6-6,0)	6.4<br>(5.6-7.5)
**Bilirubin [mg/dl]**<br>(normal 0,3-1,21)	0.3<br>(0.2-0.5)
**AST [U/l]**<br>(normal < 35)	16.0<br>(13.0-19.0)
**ALT [U/l]**<br>(normal < 35)	16.0<br>(13.0-22.0)
**LDH [U/l]**<br>(normal 120-247)	217<br>(198-248)
**Ferritin [µg/l]**<br>(normal 22-322)	459<br>(266-733)
**hs CRP [mg/dl]**<br>(normal, < 0,3)	0.5<br>(0.2-0.9)
**PCT [ng/ml]**<br>(normal 0-0,5)	0.3<br>(0.2-0.5)

Table shows median and interquartile range (IQR)

Abbreviation: ALT alanine aminotransferase, AST aspartate aminotransferase, BUN blood urea nitrogen, hsCRP high sensitivity C-reactive protein, LDH lactate dehydrogenase, PCT procalcitonin, WBC white blood cell count. Serum creatine was determined with enzymatic method.

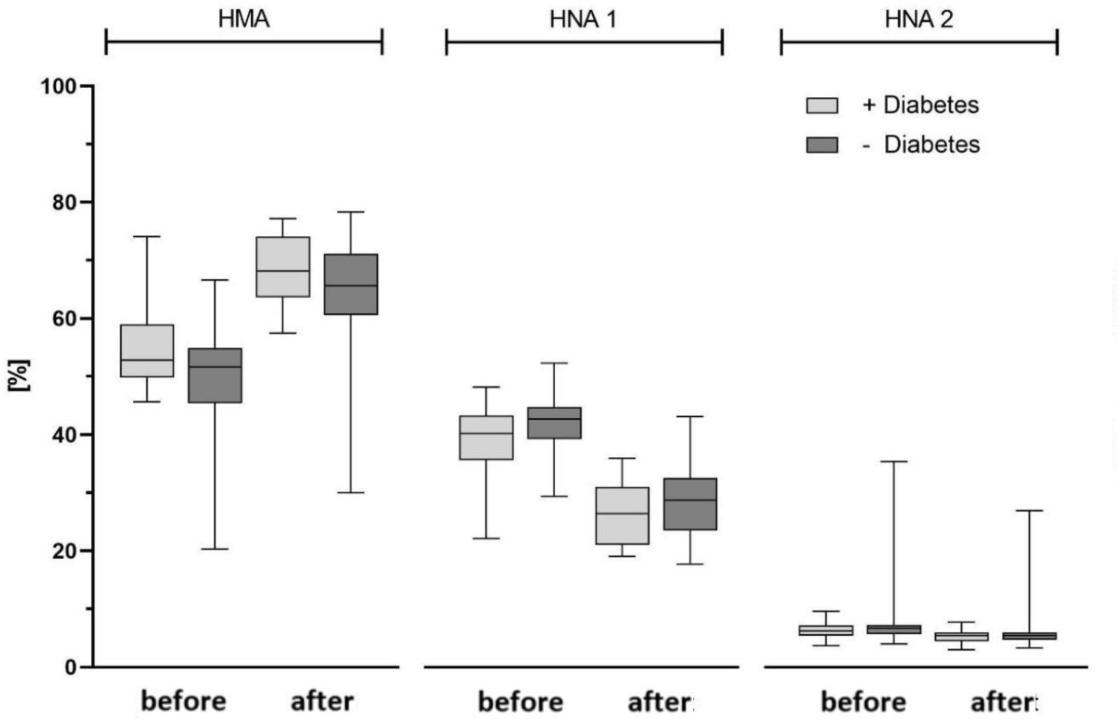

Supplement Figure 1: Albumin redox state in relation to the presence of diabetes
Boxes show human mercaptalbumin fraction (HMA), human nonmercaptalbumin-1 (HNA-1) and human nonmercaptalbumin-2 (HNA-2) before and after haemodialysis treatment. Data include n = 32 patients (diabetes subgroup n = 15, no diabetes subgroup n = 18). All patients were treated with a FX10 dialyzer. Central lines denote median values, and upper and lower borders represent 25th and 75th percentiles. The whiskers represent 5–95 percentile. Highest and lowest values are marked
